# Infrared Spectroscopy of Neutral and Cationic Benzonitrile–Methanol Binary Clusters in Supersonic Jets

**DOI:** 10.3390/molecules29122744

**Published:** 2024-06-08

**Authors:** Xianming Xiong, Yongjun Hu

**Affiliations:** MOE Key Laboratory of Laser Life Science & Guangdong Provincial Key Laboratory of Laser Life Science, Guangzhou Key Laboratory of Spectral Analysis and Functional Probes, College of Biophotonics, South China Normal University, Guangzhou 510631, China

**Keywords:** IR spectroscopy, nitrogen-containing interstellar molecules, clusters, gas phase

## Abstract

The formation of nitrogen-containing organic interstellar molecules is of great importance to reveal chemical processes and the origin of life on Earth. Benzonitrile (BN) is one of the simplest nitrogen-containing aromatic molecules in the interstellar medium (ISM) that has been detected in recent years. Methanol (CH_3_OH) exists widely in interstellar space with high reactivity. Herein, we measured the infrared (IR) spectra of neutral and cationic BN–CH_3_OH clusters by vacuum ultraviolet (VUV) photoionization combined with time-of-flight mass spectrometry. Combining IR spectra with the density functional theory calculations, we reveal that the BN–CH_3_OH intends to form a cyclic H-bonded structure in neutral clusters. However, after the ionization of BN–CH_3_OH clusters, proton-shared N···H···O and N···H···C structures are confirmed to form between BN and CH_3_OH, with the minor coexistence of H-bond and O-π structures. The formation of the proton-shared structure expands our knowledge of the evolution of the life-related nitrogen-containing molecules in the universe and provides a possible pathway to the further study of biorelevant aromatic organic macromolecules.

## 1. Introduction

In recent years, an increasing number of complex biorelevant molecules has been identified in the interstellar medium (ISM) [1,2,3,4,5]. Most of them are nitrogen-containing organic molecules [6,7,8], which are crucial constituents of biorelevant molecules [9]. How these biologically relevant molecules are generated from simpler nitrogen-containing interstellar molecules under extremely low temperatures with very intense cosmic radiation [10,11,12] and are of great interest to researchers on account of their possibility of revealing the chemical process of the universe and the evolution of life on Earth. Though a great number of studies have proposed the potential synthetic routes and formation process of large organic compounds [13,14,15] from chain nitrogen-containing molecules, there is still a lack of research on these cyclic and aromatic nitrogen-containing molecules, which need to be further investigated.

Benzonitrile (C_6_H_5_CN, BN) is one of the simplest nitrogen-containing aromatic molecules in the interstellar atmosphere. McGuire et al. first detected hyperfine-resolved transitions of benzonitrile from the molecular cloud TMC-1, in which its condition was similar to the environment of primitive Earth, with many small molecules related to life [16].

It may be a precursor for polycyclic aromatic hydrocarbon and prebiotic molecule formation, providing a relationship to the unidentified infrared bands in outer space, which have given significant concerns to researchers in recent years. It is generally accepted that BN could occur with a nucleophilic addition and electrophilic substitution reaction due to the cyano group’s ability to withdraw electrons and the phenyl ring’s conjugated system [17,18]. Additionally, methanol is the simplest molecule of alcohol, which is a significant material of polar solvents in addition to water [19]. Methanol is also a key molecule in the interstellar medium as it is ubiquitous in space [20,21], playing an essential role in the evolution mechanism of forming more complex interstellar organic molecules. Therefore, we used benzonitrile and methanol as precursors to investigate the potential reaction in nitrogenous organic macromolecules.

Ion–molecule reactions in the gas phase induced by vacuum ultraviolet are reported to be feasible to form C–C covalent bonds [22,23] and generate complex organic bio-compounds from small molecules [24], which is demonstrated by IR-VUV spectroscopy. For instance, Jiang et al. revealed that new covalent bonds formed efficiently between titanium atoms and carbon monoxide upon laser vaporization via IR-VUV photoionization spectroscopy [25]. Our previous study indicated that a new C–C covalent bond was formed in acrylonitrile dimers clusters [26], as well as in acrylonitrile and methanol clusters [27] after vacuum ultraviolet photoionization. It is worth mentioning that acrylonitrile and methanol molecules can further cause a series of Michael additions and cyclization reactions to take place and form cyclic amine structures within peptide bonds [28], which act as the main constituent of complex biorelevant molecules. In contrast with acrylonitrile, BN with the π electron cloud of aromatic benzene could take part in π hydrogen bonds (π H-bonds), cation/anion-π interactions, and π-stacking [29,30], which is of great importance for the chemistry and the stability of biological macromolecules. Fujii et al. displayed spectroscopic evidence of the “on-ring” structure in which the nonbonding electrons of the methanol directly interacted with the π orbital of the benzene in (benzene-methanol)^+^ cation clusters [31]. Dopfer et al. indicated that π-stacking occurred between the aromatic ring of BN^+^ and Ar/N_2_ moieties, while it formed a bifurcated CH··· O ionic H-bond to two adjoining CH groups and cationic clusters of benzonitrile and H_2_O clusters [32]. Therefore, we are interested in investigating intermolecular interactions between the nitrogen-containing aromatic molecules benzonitrile and methanol clusters and possible ion–molecule reactions between them.

In order to investigate the possible intermolecular interactions between benzonitrile and methanol, IR spectra combined with theoretical calculations of neutral and cationic BN–CH_3_OH gaseous clusters have been carried out. The proton-shared structures formed between benzonitrile and methanol are indicated by the IR spectral features with ab initio calculations.

## 2. Results

### 2.1. Time-of-Flight Mass Spectrum

**Figure 1:**
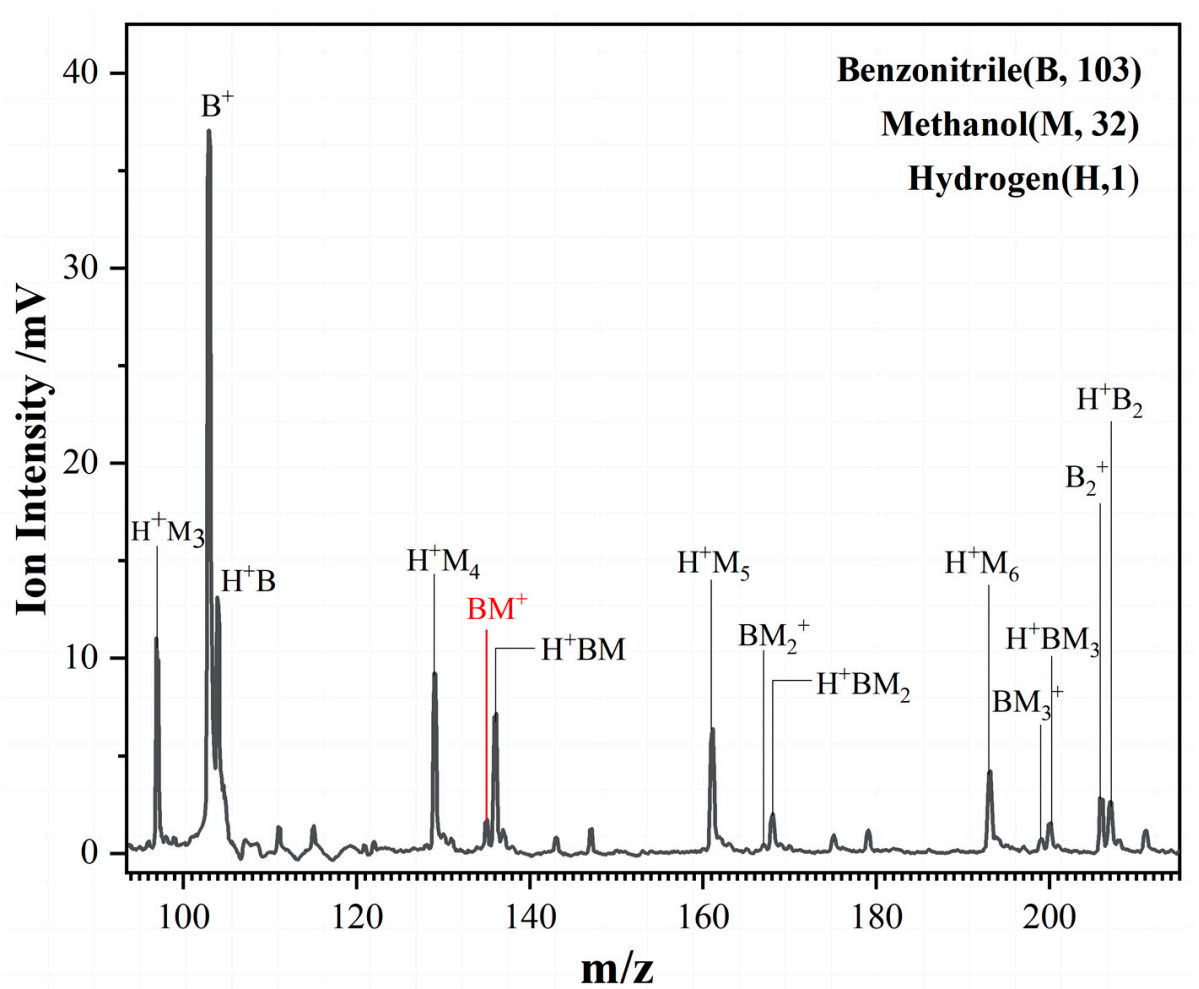
TOF mass spectrum of BN–CH_3_OH mixture clusters in molecular beams ionized by 118 nm light. The target ion channels we measured were marked in red stick with red text in the spectrum. The assignation of other clusters is also annotated in the figure.

Gaseous BN–CH_3_OH clusters generated in the supersonic expansion and ionized by 118 nm VUV light, subsequently analyzed using a time-of-flight (TOF) mass spectrometer equipped with a microchannel plate (MCP) detector, enable clusters of different sizes to be ultimately mass-selected. The mass spectrum of the VUV-ionized BN–CH_3_OH cluster ions is shown in Figure 1. The single photon energy at a wavelength of 118 nm is 10.5 eV, with the gas phase ionization energy of benzonitrile being 9.7 eV and that of methanol being 10.85 eV. Consequently, the 118 nm single photon is sufficient to ionize benzonitrile but is inadequate for the ionization of methanol monomers. As a result, signals of benzonitrile monomers are observed in the mass spectrum, whereas no signals of methanol monomers are detected. However, due to a reduction in ionization energy upon cluster formation, signals from protonated methanol polymers, benzonitrile dimers, and clusters combining a single proton (protonation) of benzonitrile (H^+^B) and its protonation dimers are detected, as well as a variety of mixed clusters, such as BN–CH_3_OH (BM^+^), H^+^BM, BN–(CH_3_OH)_2_ (BM_2_^+^), H^+^BM_2_, BN–(CH_3_OH)_3_ (BM_3_^+^) and H^+^BM_3_. The mass number of the benzonitrile molecule is 103, and that of the CH_3_OH molecule is 32. The IR spectra of the selected cluster were measured by observing the channels of (BN–CH_3_OH) (*m*/*z* = 135).

### 2.2. IR Spectra of Neutral BN–CH_3_OH Clusters

**Figure 2:**
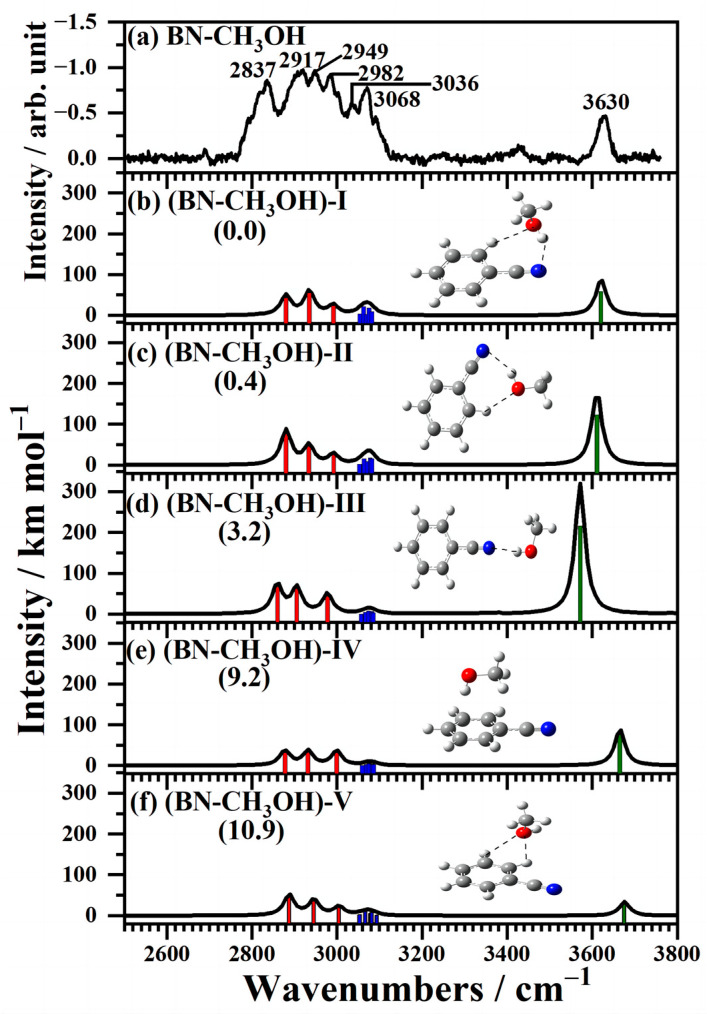
(**a**) Experimental and (**b**–**f**) calculated IR spectra of neutral (BN–CH_3_OH) in the 2500−3800 cm^−1^ region. Calculated spectra with a scaled factor of 0.96 under B3LYP-D3(BJ)/aug-cc-pVDZ method, and the convoluted spectra generated by a Lorentzian line shape function with a width of 15 cm^−1^ (FWHM). The predicted structures and relative energy at the zero-point vibrational level (the number in parentheses in kJ mol^−1^) are also shown. The red, blue, and green sticks represent the CH stretching vibration of C (*sp*^3^)–H and C (*sp*^2^)–H and OH stretching vibration, respectively.

The observed and calculated IR spectra of neutral BN–CH_3_OH clusters in the CH and OH region of 2500−3800 cm^−1^ is illustrated in Figure 2 and the predicted structures of BN–CH_3_OH isomers with relative energy at the zero-point vibrational level are inserted. The red, blue, and green sticks represent the CH stretching vibration of C (*sp*^3^)–H and C (*sp*^2^)–H and OH stretching vibration, respectively. Seven distinct bands are exhibited at 2837, 2917, 2949, 2982, 3036, 3068 and 3630 cm^−1^. The region between 2800 and 3100 cm^−1^ features a group of adjacent bands, which correspond to the stretching vibrations of the CH bond of CH_3_OH and benzonitrile compared with the stretching vibration of the CH of CH_3_OH and benzonitrile appearing at 2850–2960 cm^−1^ and 3000–3100 cm^−1^, respectively [33,34]. For the free OH stretching vibration of (CH_3_OH)_3_^+^ emerging at 3655 cm^−1^ [35], the 3630 cm^−1^ band is attributed to the H-bonded OH stretching vibration. 

Nine isomers of the BN–CH_3_OH clusters were found, and five stable structures with the calculated infrared spectra are displayed in Figure 2b–f in comparison with the experimental spectra in Figure 2a. Other neutral structures with relatively higher energies are also shown in Figure S1. Meanwhile, the simulated structures are shown. C–H···O and O–H···N intermolecular H-bonds are generated in structures (BN–CH_3_OH)-I and II, which constitute H-bonded cyclic networks. It is worth mentioning that in the structure (BN–CH_3_OH)-I, the methyl group of CH_3_OH is oriented nearly perpendicular to the plane of the benzonitrile, whereas in the (BN–CH_3_OH)-II, CH_3_OH and the benzonitrile are almost coplanar. In the structure (BN–CH_3_OH)-III, an intermolecular O–H···N linear H-bond was generated, and CH_3_OH acted as an H-donor. In the structure (BN–CH_3_OH)-IV, CH_3_OH is above the plane of the benzonitrile, interacting with the π orbital of the benzonitrile. However, a bifurcated CH···O hydrogen bond for two adjoining CH groups of benzonitrile and CH_3_OH clusters formed in the (BN–CH_3_OH)-V, which showed relatively higher energy.

The little differences in relative energies (zero-point energy) for the five structures are 0.0, 0.4, 3.2, 9.2, and 10.9 kJ mol^−1^, respectively. The predicted IR spectra of (BN–CH_3_OH)-I and II agree well with the experiment. The vibrational frequencies of the simulated structures and their assignment are noted in Table S1. In consideration of the observed bands of (BN–CH_3_OH)-III, IV, and V structures, they are less likely to exist as the low-frequency and high-intensity OH stretching vibration of (BN–CH_3_OH)-III and high-frequency OH stretching vibration of (BN–CH_3_OH)-IV and V. It is clear that the infrared spectra of (BN–CH_3_OH)-I and II agree well with the experiment spectrum. For (BN–CH_3_OH)-I with the lowest relative energy of all, we reveal that it is dominant in (BN–CH_3_OH) clusters. The results are similar to the study of BNCH_3_OH clusters by fluorescence-detected Raman, infrared spectroscopies, and high-resolution fluorescence excitation spectra in the gas phase [36,37], which showed a structure where CH_3_OH was H bonded to the CN group and to the adjacent hydrogen atom of BN. However, in the ground-state structures, the CH_3_OH molecule is H-bonded to the nitrogen atom of cyano through the H atom of its hydroxyl group [38] with the TDDFT calculation. Nevertheless, (BN–CH_3_OH)-II is only 0.4 kJ mol^−1^ higher than (BN–CH_3_OH)-I in its energy level and, with similar cyclic H-bond structures, (BN–CH_3_OH)-II might also be of existence in the experiment.

### 2.3. IR Spectra of Cationic (BN–CH_3_OH)^+^ Clusters

**Figure 3:**
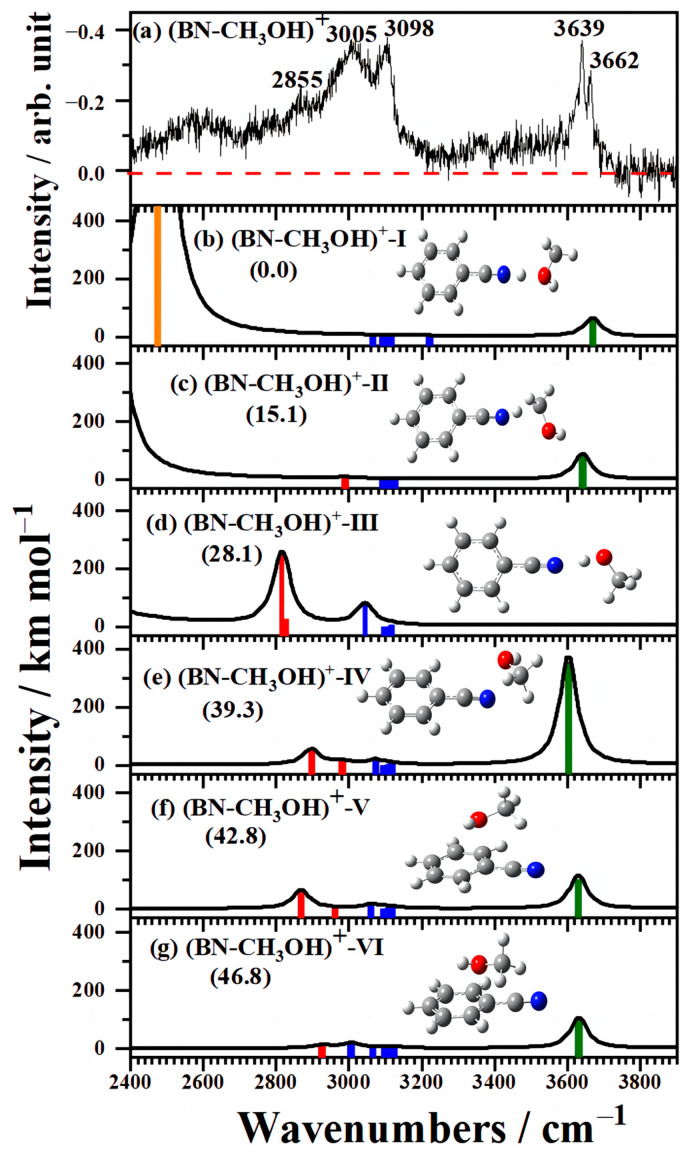
(**a**) Experimental and (**b**–**g**) calculated IR spectra of (BN–CH_3_OH)^+^ in the 2400−3900 cm^−1^ region. Calculated spectra with a scaled factor of 0.967 under the B3LYP-D3(BJ)/aug-cc-pVDZ method and the convoluted spectra generated by a Lorentzian line shape function with a width of 30 cm^−1^ (FWHM). The predicted structures with relative energy at the zero-point vibrational level (the number in parentheses in kJ mol^−1^) are also shown. The orange, red, blue, and green sticks represent the proton-shared NH stretching vibration, CH stretching vibration of C (*sp^3^*)–H and C (*sp^2^*)–H and OH stretching vibration, respectively.

Figure 3a displays the experiment infrared spectrum of (BN–CH_3_OH)^+^ in the 2400–3900 cm^−1^ region consisting of two sharp peaks at 3629 cm^−1^ and 3662 cm^−1^ and three broad bands at 2855, 3005 and 3098 cm^−1^ associated with the CH-stretching vibration. In addition, a broad band extended from 3500 cm^−1^ to the lower frequency region in the spectrum of (BN–CH_3_OH)^+^. Similar to the cationic acrylonitrile and NH_3_ [39] and previous results of strong H-bonded XH (X = N, O) band [40,41] structures, the broad band may be caused by the strong intermolecular interactions between the H atom of CH_3_OH and electron-deficient N atom of benzonitrile. It is worth mentioning that there are two splitting bands of OH stretching vibrations at 3629 cm^−1^ and 3662 cm^−1^, which indicate the coexistence of two isomers in (BN–CH_3_OH)^+^, referring to the IR spectrum results of cationic benzene and CH_3_OH clusters [31]. The broad band with three peaks at 2855, 3005, and 3098 cm^−1^ is attributed to the CH-stretching vibrations of CH_3_OH and benzonitrile. In contrast to the spectrum of (AN–CH_3_OH) ^+^ [27], there is no strong band at ∼3500 cm^−1^, which may indicate the absence of intermolecular proton transfer without the N–H covalent bond formation in (BN–CH_3_OH)^+^ clusters.

In order to further investigate the detailed process of the ion–molecular reaction in (BN–CH_3_OH)^+^ clusters, the infrared spectra of predicted structures of (BN–CH_3_OH)^+^ were simulated in comparison with the experiment spectra. More than 30 structures of (BN–CH_3_OH)^+^ were found, including different types of weak intermolecular interactions and new bond formations. The six most stable structures of weak intermolecular interactions are shown in Figure 3b–g, and other structures are also shown in Figures S2 and S3. The isomers of added products and new C–C bond formations are shown in the supporting information in Figures S4 and S5. The red, blue, and green sticks represent the CH stretching vibration of C (*sp*^3^)–H and C (*sp*^2^)–H, and the OH stretching vibration, respectively. The six isomers were divided into three types according to different structural features. Three of them formed strong proton-shared structures, and one formed an H-bond in the clusters, while two others formed intermolecular interactions by O–π bonds. Considering that the calculated intense NH stretching vibration at ∼3400 cm^−1^ did not appear in the experimental spectra, the isomers that went through proton transfer and new C–C covalent bond formations may not exist in (BN–CH_3_OH)^+^ clusters. In the most stable structure (BN–CH_3_OH)^+^-I, the proton of CH_3_ is shared by N and O atoms, while the proton of CH_3_ is shared by the N and C atoms of CH_3_OH in the structure (BN–CH_3_OH)^+^-Ⅱ. In contrast, proton-shared N···H···O structures are formed between the benzonitrile and OH of CH_3_OH in the structure (BN–CH_3_OH)^+^-Ⅲ. In the structure (BN–CH_3_OH)^+^-IV, CH_3_OH is above benzonitrile and contacts each other via a weak O–H···N intramolecular H-bond. The nonbonding electrons of CH_3_OH directly interact with the π orbital of the benzonitrile cation moiety in (BN–CH_3_OH)^+^-V and Ⅵ. The relative energies of the predicted structures are 0.0, 15.1, 28.1, 39.3, 42.8, and 46.8 kJ mol^−1^, respectively.

The simulated infrared spectra of the comparatively stable six structures of (BN–CH_3_OH)^+^ by the B3LYP-D3(BJ)/aug-cc-pVDZ method are displayed in Figure 3b–g and compared with the experiment spectrum in Figure 3a. The vibration frequencies and their assignment are noted in Table S2. In addition, the simulated infrared spectra under M06-2x/aug-cc-pVDZ and MP2/aug-cc-pVDZ and relative energies are provided in the supporting information in Figures S6 and S7, and Table S3, which both show similar results to the B3LYP method. The broad band appearing in the observed spectrum was attributed to the stretching vibrations of proton-shared NH in structures (BN–CH_3_OH)^+^-I and Ⅱ. The discrepancy between the experimental and computational intensities and frequencies of proton-shared NH in structures (BN–CH_3_OH)^+^-I and (BN–CH_3_OH)^+^-Ⅱ may be due to the strongly anharmonic proton motion of N···H···O and N···H···C structures compared to the computed harmonic frequency, which was reported in ClH/NH_3_ and HBr/pyridine [42,43], which appears to be a broad band extended from 3500 cm^−1^ to the lower frequency region. The bands appearing at 2855, 3005, and 3098 cm^−1^ are associated with the CH bond stretching vibrations of C (*sp*^3^)–H in CH_3_OH and C (*sp*^2^)–H in benzonitrile, respectively. The simulated IR spectra of the six structures are similar in the CH-stretching region. In addition, the spectral features of cationic complexes in Figure 3 by M06-2x/aug-cc-pVDZ and MP2/aug-cc-pVDZ computational methods are similar to the spectra under the B3LYP-D3(BJ)/aug-cc-pVDZ method, and the two most stable structures are (BN–CH_3_OH)^+^-I and (BN–CH_3_OH)^+^-Ⅱ, as also shown by all the three methods in the supporting information Table S3. The bands of the OH stretching vibration of the structures (BN–CH_3_OH)^+^-I and Ⅱ agree extremely well with the experiment spectrum, which shows the splitting of the OH stretch band at 3629 cm^−1^ and 3662 cm^−1^. The experimental and computational intensities show differences in the CH and OH stretching vibration region, as the spectra that we obtained are vacuum ultraviolet ionization infrared dissociation action spectroscopy, which is different from the infrared absorption spectrum by calculation. The dissociation efficiency of absorption infrared light in the CH region may be higher than that of the OH region, resulting in the CH stretching vibration intensity showing a discrepancy compared to the computational spectra. Therefore, we reveal that (BN–CH_3_OH)^+^-I and Ⅱ is the major stable isomer of (BN–CH_3_OH)^+^ and proton-shared N···H···O and N···H···C structures formed between cationic benzonitrile and CH_3_OH.

### 2.4. Energy Diagram for the Isomerization Reaction of (BN–CH_3_OH)^+^

**Figure 4:**
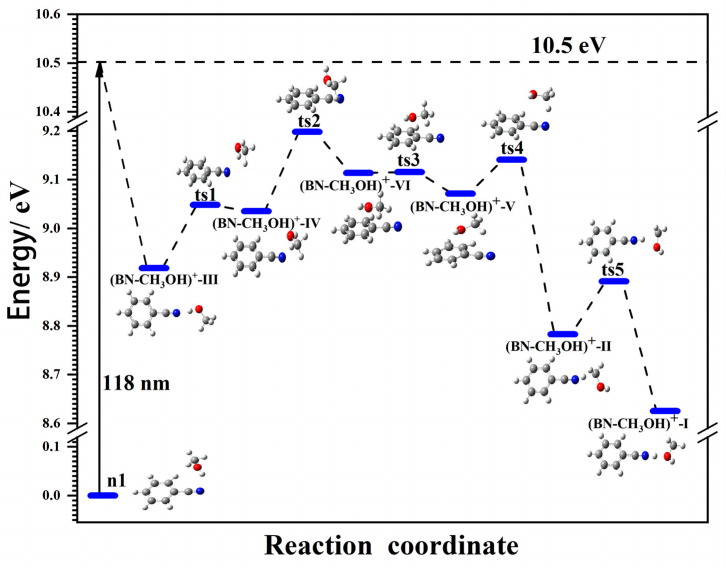
Energy diagram for the isomerization process of (BN–CH_3_OH)^+^ under 118 nm (10.5 eV) VUV light ionization from the most stable neutral structures **n1**. The energies in the diagram were calculated under the B3LYP-D3(BJ)/aug-cc-pVDZ method.

To gain a deeper understanding of the process of the possible ion–molecule reactions in (BN–CH_3_OH) upon ionization, we conducted the reaction path search by using the global reaction route mapping (GRRM) reaction path search calculations [44,45,46] method. Figure 4 shows the reaction energy diagram of the (BN–CH_3_OH) cluster upon ionization from the most stable neutral structure **n1**.

On the basis of the reaction diagram, upon the ionization of the **n1** structure, the proton of the OH bond of CH_3_OH transfers to the N atom of benzonitrile followed by (BN–CH_3_OH)^+^-Ⅲ formation; the bands at 2855 cm^−1^ also indicate that the structure may form after the ionization of the neutral **n1** structure. It is worth mentioning that the two most stable neutral structures (BN–CH_3_OH)-Ⅰ (**n1**) and (BN–CH_3_OH)-Ⅱ both form structure (BN–CH_3_OH)^+^-Ⅲ after ionization. On the reaction pathway, CH_3_OH in (BN–CH_3_OH)^+^-Ⅲ firstly undergoes movement to the top of the benzonitrile plane, followed by O–H···N H-bond formation to gain the structure (BN–CH_3_OH)^+^-IV via a barrier of **ts1**. Following isomerization through the transition states **ts2** and **ts3**, respectively, the isomer (BN–CH_3_OH)^+^-Ⅵ and V, both with intermolecular interactions by O-π bonds, are formed. After the formation of the (BN–CH_3_OH)^+^-V structure, the CH_3_ of CH_3_OH approaches the cyano of benzonitrile to form the proton-shared N···H···C structure (BN–CH_3_OH)^+^-Ⅱ through the transition state **ts4**. The relative energy of (BN–CH_3_OH)^+^-Ⅱ is much lower in comparison with other structures due to the intermolecular interactions between benzonitrile and CH_3_OH. Finally, with the rotation of carbon-centered radical (i.e.,• CH_2_OH) moieties [47,48], the most stable (BN–CH_3_OH)^+^-Ⅰ structure with proton-shared N···H···O is formed via transition state **ts5**. In addition, the comparatively lower energy barrier (**ts5**) of the most stable (BN–CH_3_OH)^+^-Ⅰ structures reveals that (BN–CH_3_OH)^+^-Ⅰ is the dominant product of (BN–CH_3_OH)^+^ in the supersonic jet.

In general, proton-shared N···H···O and N···H···C structures are formed between benzonitrile and methanol. This is in contrast to the (acrylonitrile–methanol)^+^ [27] and (benzene–methanol)^+^ [31] clusters, in which new C–C covalent bonds or an O-π structure forms, respectively. It may be caused by the different structure features and ionization sequence of them. The ionization energy of methanol (10.84 eV) is higher than that of benzonitrile (9.73 eV) and benzene (9.24 eV), while it is a little lower in contrast to acrylonitrile (10.91 eV). Therefore, electrons are primarily ejected from methanol, and subsequently, methanol transforms as a Michael donor [49], resulting in electrostatic attraction and the formation of a C–C covalent bond through the Michael addition reaction in (acrylonitrile–methanol)^+^ clusters. Conversely, in the case of benzonitrile and benzene, electrons are preferentially ejected, and the stability conferred by the electron-rich π-bond conjugated system of the benzene ring inhibits the formation of electrostatic interactions and new C–C covalent bonds with methanol molecules. On the other hand, it tends to form the proton-shared structure in (BN–CH_3_OH)^+^ due to the cyano group’s ability to withdraw protons, while only H-bond formation occurs in (benzene–methanol)^+^.

## 3. Experimental and Computational Methods

### 3.1. Experimental Method

The neutral and cationic BN–CH_3_OH clusters were both size-selected through a time-of-flight (TOF) mass spectrometer. Vacuum ultraviolet (VUV) light provided the ionization source combined with a tunable IR beam for dissociation. The detailed experimental method and instruments were introduced earlier [50,51,52,53]. In short, benzonitrile and methanol clusters were produced by the supersonic expansion of a gas mixture with helium through a pulse valve. To measure the infrared spectra of neutral and cationic clusters, tunable IR light was generated by an optical parametric oscillator/amplifier (OPO/OPA) system and was introduced 50 ns before or 200 ns after VUV light. Various clusters were detected using a microchannel plate (MCP). By changing the frequency of IR light via the OPO/OPA system, the infrared spectra of the selected clusters were measured by observing the ion mass signal intensities.

In total, 355 nm of light generated by the Nd/YAG laser (INDI-40 SPECTRA-PHYSICS) produced 118 nm of VUV light via the frequency tripling of the third harmonic in argon and xenon mixture gas (Xe/Ar = 1:10) at ∼200 Torr. Tunable IR radiation is generated through the OPO/OPA system and serves IR power in the range of 4 to 12 mJ with wavenumbers ranging from 2400 to 3900 cm^−1^ [54].

### 3.2. Computational Details

Structure optimization, simulated IR spectra, and relative energy were performed by the Gaussian 16 package [55,56]. All stable structures and transition states were obtained from GRRM reaction path search calculations [44,45,46] with the B3LYP-D3(BJ)/aug-cc-pVDZ method. The computational method of B3LYP was the same as the study of (AN–CH_3_OH)^+^, (BN–H_2_O)^+^, and (BZ–CH_3_OH)^+^ [27,31,32], the spectra of which were well reproduced compared to the experiment spectra. Additionally, the cationic complexes in Figure 3 were recalculated at the M06-2x/aug-cc-pVDZ and MP2/aug-cc-pVDZ levels, with relative energies and vibration frequency calculations. The calculated frequencies of neutral and cation gas clusters were scaled by suitable factors of 0.960 and 0.967, respectively. These were determined by referring to the (BN–H_2_O)^+^ and (BZ–CH_3_OH)^+^ clusters [31,32].The calculated cation spectra display the resulting bar spectra convolved with the Lorentzian line shape function with a width of 30 cm^−1^ and the neutral spectra with 15 cm^−1^ widths, respectively.

## 4. Conclusions

In summary, the IR spectra of neutral and cationic BN–CH_3_OH clusters in a supersonic jet were measured by vacuum ultraviolet ionization. With the combination of the density functional theory (DFT) calculations, it was revealed that BN–CH_3_OH interacts by C–H···O and O–H···N intermolecular H-bonds to form cyclic network structures in neutral clusters. After vacuum ultraviolet photoionization, however, the results indicated that proton-shared N···H···O and N···H···C structures formed between benzonitrile and CH_3_OH. Furthermore, the proton-shared cation can be generated through a series of isomerization reactions of proton movement, H-bonds, and O-π structure formation after ionization of the neutral BN–CH_3_OH. This result of proton-shared structure formation is different from the (acrylonitrile–methanol)^+^ and (benzene–methanol)^+^ clusters, which form new C-C covalent bonds and the O-π structure, respectively. The IR spectrum of (BN–CH_3_OH)^+^ is measured to study predicted structures and possible reaction processes between BN and CH_3_OH with a detailed analysis of the IR spectra combined with DFT calculations. These results may greatly expand our knowledge of the process of prebiotic organic macromolecule formation from nitrogen-containing interstellar molecules in the cosmic atmosphere.

## Data Availability

Data are contained within the article and Supplementary Materials.

## Supplementary Materials

The following supporting information can be downloaded at: https://www.mdpi.com/article/10.3390/molecules29122744/s1. Figure S1: Calculated IR spectra of other neutral structures of (BN–CH_3_OH) in the 2500−3800 cm^−1^ region; Figures S2–S5: Calculated IR spectra of other structures of (BN–CH_3_OH)^+^ in the 2400−3900 cm^−1^ region; Figure S6: Calculated IR spectra of (BN–CH_3_OH)^+^ clusters in Figure 3 in the 2400−3900 cm^−1^ region under the M06-2x/aug-cc-pVDZ method; Figure S7: Calculated IR spectra of (BN–CH_3_OH)^+^ clusters in Figure 3 in the 2400−3900 cm^−1^ region under the MP2/aug-cc-pVDZ method; Table S1: Comparison of observed and scaled harmonic frequencies of (BN–CH_3_OH) and their assignments; Table S2: Comparison of observed and scaled harmonic frequencies of (BN–CH_3_OH)^+^ and their assignments; Table S3: Comparison of relative energies of (BN–CH_3_OH)^+^ clusters in Figure 3 under three different methods.
