# Supplementary material for: Infrared Spectroscopy of Neutral and Cationic Benzonitrile–Methanol Binary Clusters in Supersonic Jets
Source: Molecules. 2024 Jun 8;29(12):2744. doi: 10.3390/molecules29122744 (PMC11206090; doi:10.3390/molecules29122744)
Supplement: Supplementary file 1 [file molecules-29-02744-s001.zip › molecules-3029332-supplementary.pdf]

Electronic supplementary information (ESI)

Infrared Spectroscopy of Neutral and Cationic Benzonitrile-Methanol Binary Clusters in Supersonic Jet

Xianming Xiong and Yongjun Hu *

* Correspondence: yjhu@scnu.edu.cn (Y.H.)

AFFILIATIONS

MOE Key Laboratory of Laser Life Science & Guangdong Provincial Key Laboratory of Laser Life Science, Guangzhou Key Laboratory of Spectral Analysis and Functional Probes, College of Biophotonics, South China Normal University, Guangzhou 510631, China

AUTHOR INFORMATION

***Corresponding Author**

E-mail: yjhu@scnu.edu.cn (Y.H.)

Telephone: (+86-20)8521-1920 EXT 8713. Fax: (+86-20) 8521-6052.

Table of Contents

Figure S1. Calculated IR spectra of other neutral structures of (BN-CH_3OH) in the 2500−3800 cm^{-1} region.

Figure S2. Calculated IR spectra of other structures of (BN-CH_3OH)$^+$ in the 2400−3900 cm^{-1} region.

Figure S3. Calculated IR spectra of other structures of (BN-CH_3OH)$^+$ in the 2400−3900 cm^{-1} region.

Figure S4. Calculated IR spectra of other structures of (BN-CH_3OH)$^+$ in the 2400−3900 cm^{-1} region.

Figure S5. Calculated IR spectra of other structures of (BN-CH_3OH)$^+$ in the 2400−3900 cm^{-1} region.

Figure S6. Calculated IR spectra of (BN-CH_3OH)$^+$ clusters in Figure 3 in the 2400−3900 cm^{-1} region under M06-2x/aug-cc-pVDZ method.

Figure S7. Calculated IR spectra of (BN-CH_3OH)$^+$ clusters in Figure 3 in the 2400−3900 cm^{-1} region under MP2/aug-cc-pVDZ method.

Table S1. Comparison of observed and scaled harmonic frequencies of (BN-CH_3OH) and their assignments.

Table S2. Comparison of observed and scaled harmonic frequencies of (BN-CH_3OH)$^+$ and their assignments.

Table S3. Comparison of relative energies of (BN-CH_3OH)$^+$ clusters in Figure 3 under different three methods.

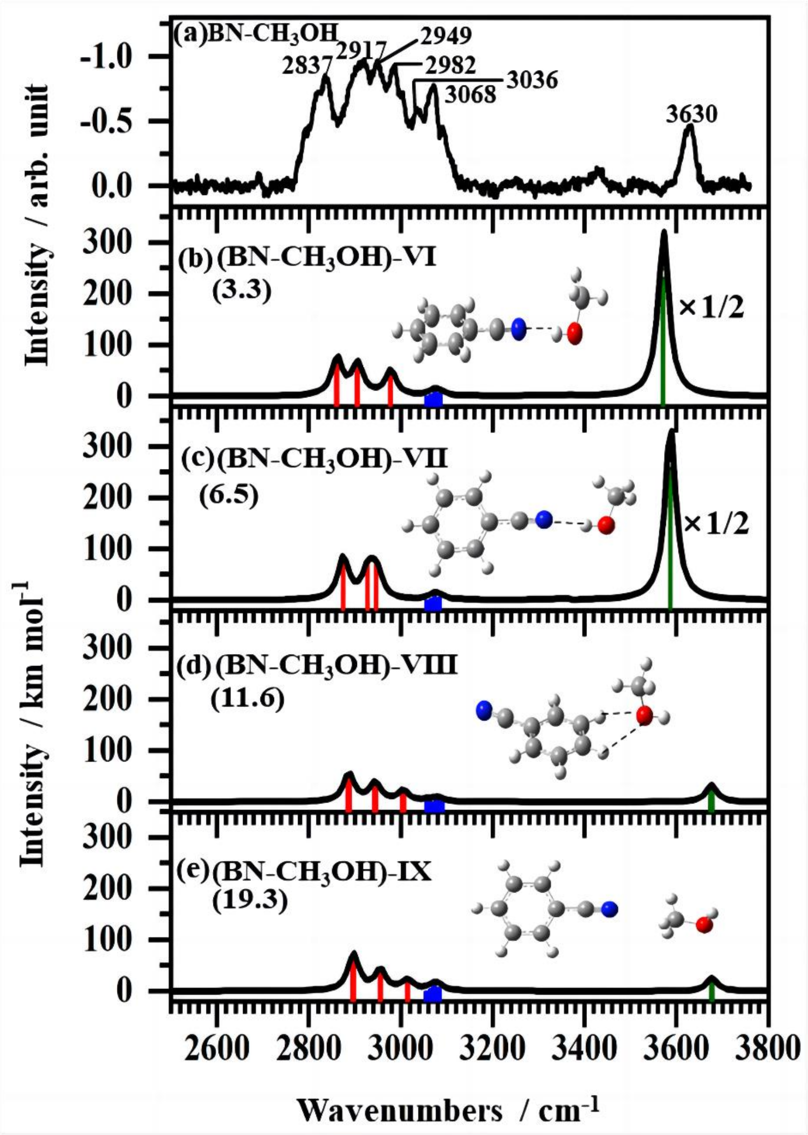

Figure S1. (a) Experimental and (b)-(e) calculated IR spectra of neutral (BN-CH_3OH) in the 2500−3800 cm^{-1} region. Calculated spectra with a scaled factor of 0.96 under B3LYP-D3(BJ)/aug-cc-pVDZ method, and the convoluted spectra generated by a Lorentzian line shape function with a width of 15 cm^{-1} (FWHM). The predicted structures and relative energy at the zero point vibrational level (the number in parentheses in kJ mol^{-1}) are also shown. The red, blue and green sticks represent the CH stretching vibration of C (sp^3)–H and C (sp^2)–H, and OH stretching vibration, respectively.

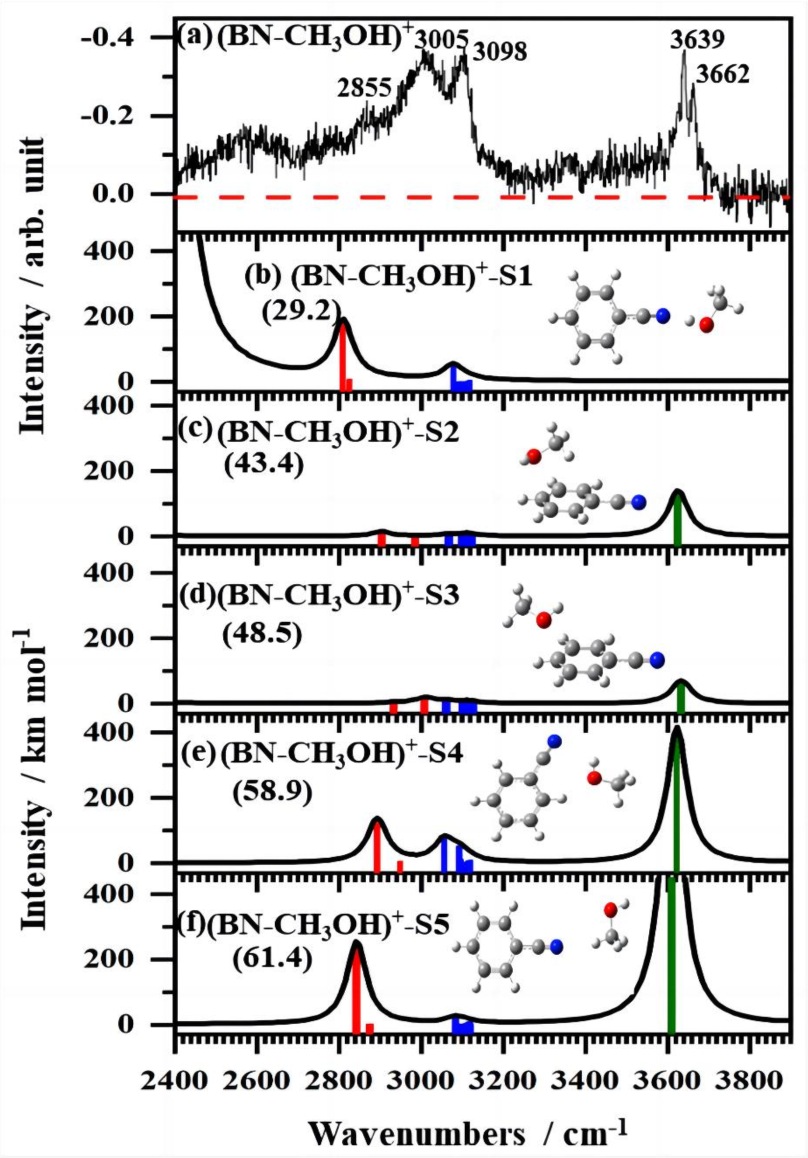

Figure S2. (a) Experimental and (b)-(f) calculated IR spectra of $(BN\text{-}CH_3OH)^+$ in the 2400−3900 cm^{-1} region. Calculated spectra with a scaled factor of 0.967 under B3LYP-D3(BJ)/aug-cc-pVDZ method, and the convoluted spectra generated by a Lorentzian line shape function with a width of 30 cm^{-1} (FWHM). The predicted structures with relative energy at the zero point vibrational level (the number in parentheses in kJ mol^{-1}) are also shown. The red, blue and green sticks represent the CH stretching vibration of C (sp^3)–H and C (sp^2)–H, and OH stretching vibration, respectively.

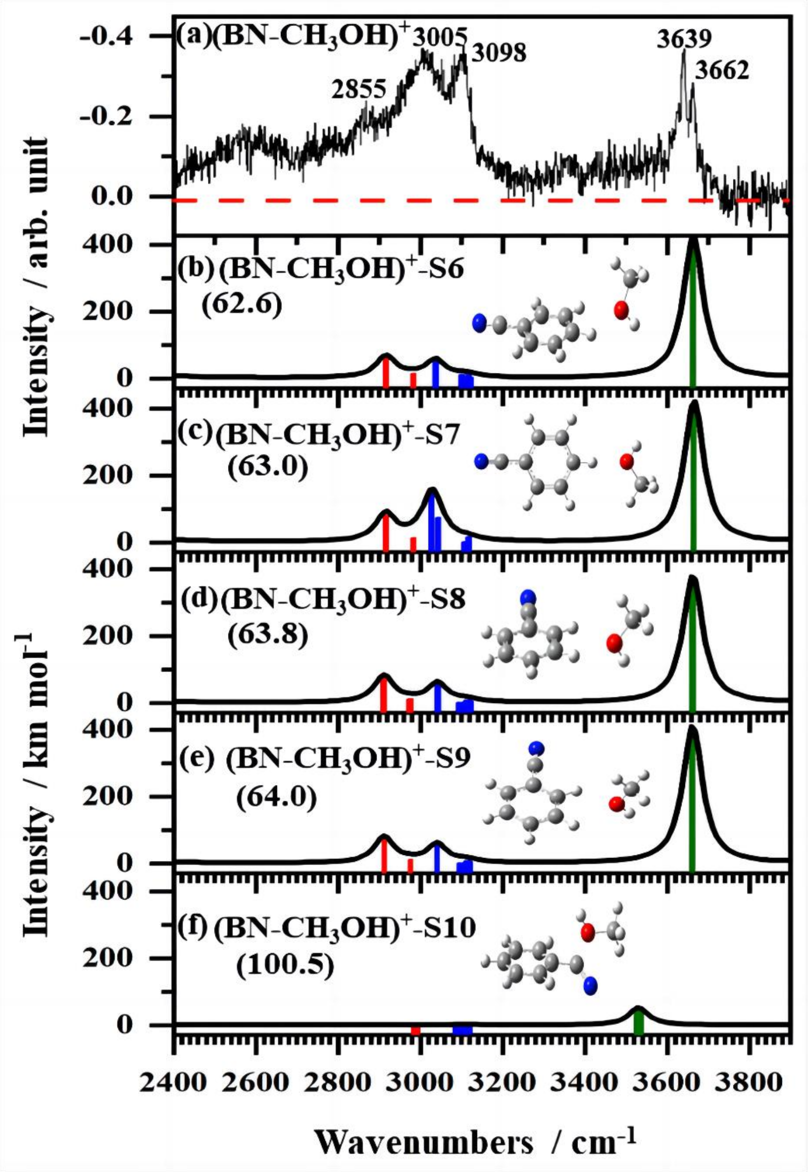

Figure S3. (a) Experimental and (b)-(f) calculated IR spectra of $(BN\text{-}CH_3OH)^+$ in the 2400−3900 cm^{-1} region. Calculated spectra with a scaled factor of 0.967 under B3LYP-D3(BJ)/aug-cc-pVDZ method, and the convoluted spectra generated by a Lorentzian line shape function with a width of 30 cm^{-1} (FWHM). The predicted structures with relative energy at the zero point vibrational level (the number in parentheses in kJ mol^{-1}) are also shown. The red, blue and green sticks represent the CH stretching vibration of C (*sp*^{3})–H and C (*sp*^{2})–H, and OH stretching vibration, respectively.

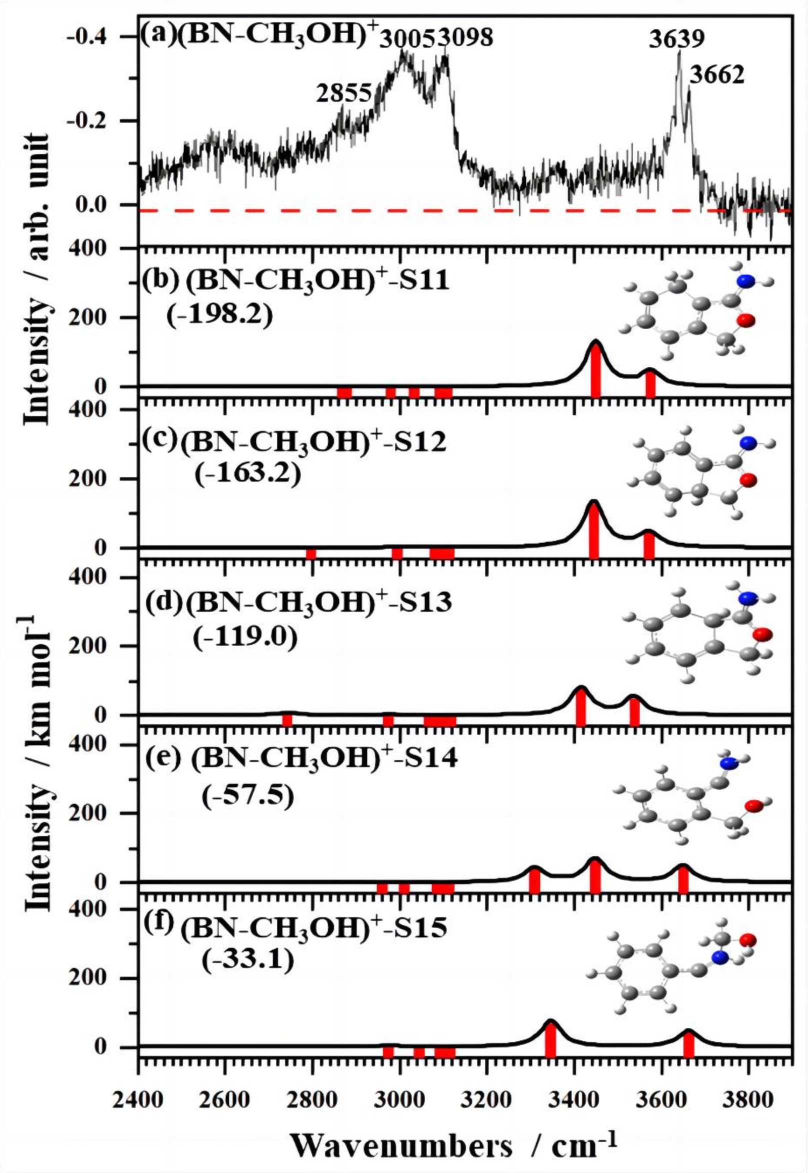

Figure S4. (a) Experimental and (b)-(f) calculated IR spectra of $(BN\text{-}CH_3OH)^+$ in the 2400−3900 cm^{-1} region. Calculated spectra with a scaled factor of 0.967 under B3LYP-D3(BJ)/aug-cc-pVDZ method, and the convoluted spectra generated by a Lorentzian line shape function with a width of 30 cm^{-1} (FWHM). The predicted structures with relative energy at the zero point vibrational level (the number in parentheses in kJ mol^{-1}) are also shown.

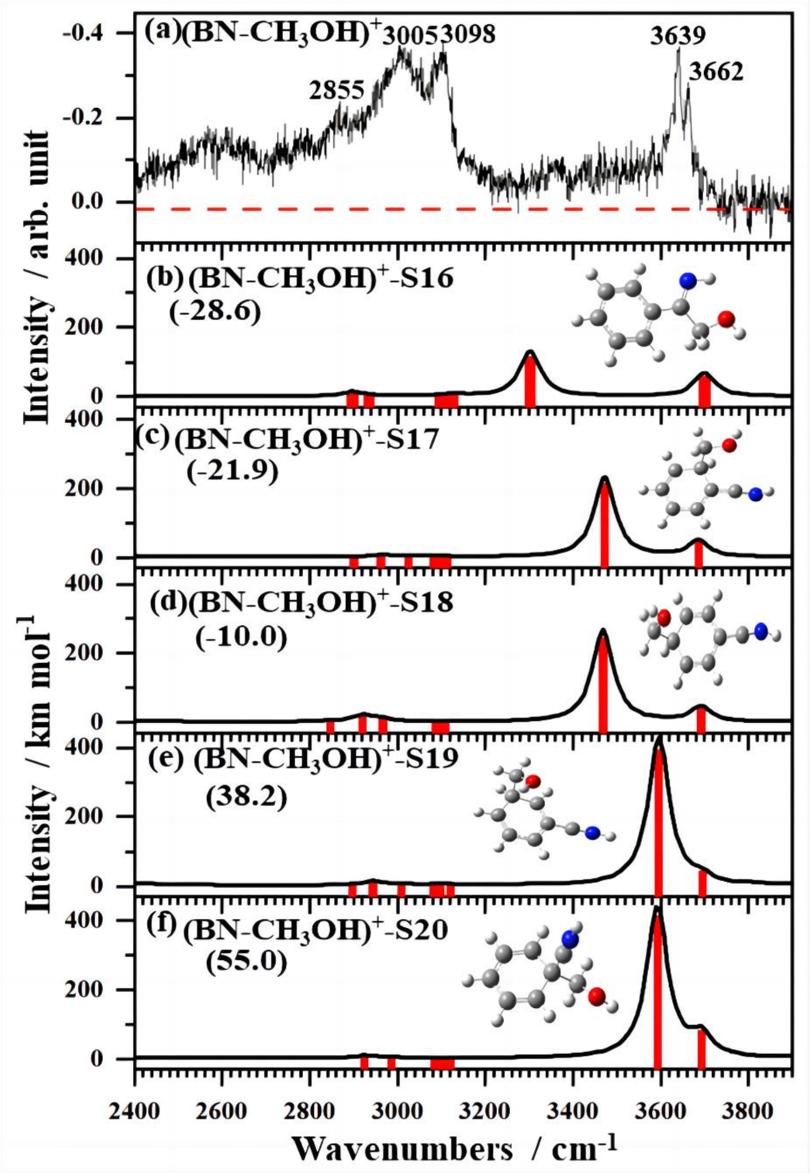

Figure S5. (a) Experimental and (b)-(f) calculated IR spectra of $(BN\text{-}CH_3OH)^+$ in the 2400−3900 cm^{-1} region. Calculated spectra with a scaled factor of 0.967 under B3LYP-D3(BJ)/aug-cc-pVDZ method, and the convoluted spectra generated by a Lorentzian line shape function with a width of 30 cm^{-1} (FWHM). The predicted structures with relative energy at the zero point vibrational level (the number in parentheses in kJ mol^{-1}) are also shown.

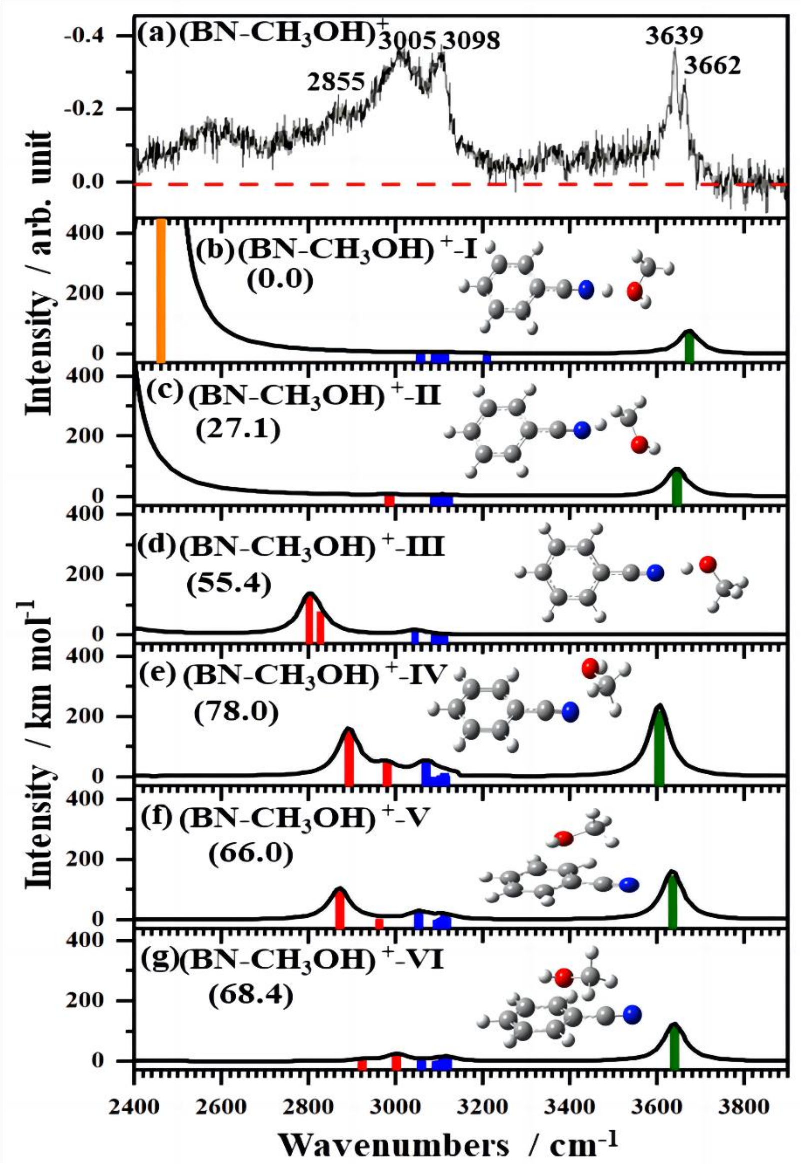

Figure S6. (a) Experimental and (b)-(g) calculated IR spectra of $(BN\text{-}CH_3OH)^+$ in the 2400−3900 cm^{-1} region. Calculated spectra with a scaled factor of 0.96 under M06-2x/aug-cc-pVDZ method, and the convoluted spectra generated by a Lorentzian line shape function with a width of 30 cm^{-1} (FWHM). The predicted structures with relative energy at the zero point vibrational level (the number in parentheses in kJ mol^{-1}) are also shown. The orange, red, blue and green sticks represent the proton-shared NH stretching vibration, CH stretching vibration of C (sp^3)–H and C (sp^2)–H, and OH stretching vibration, respectively.

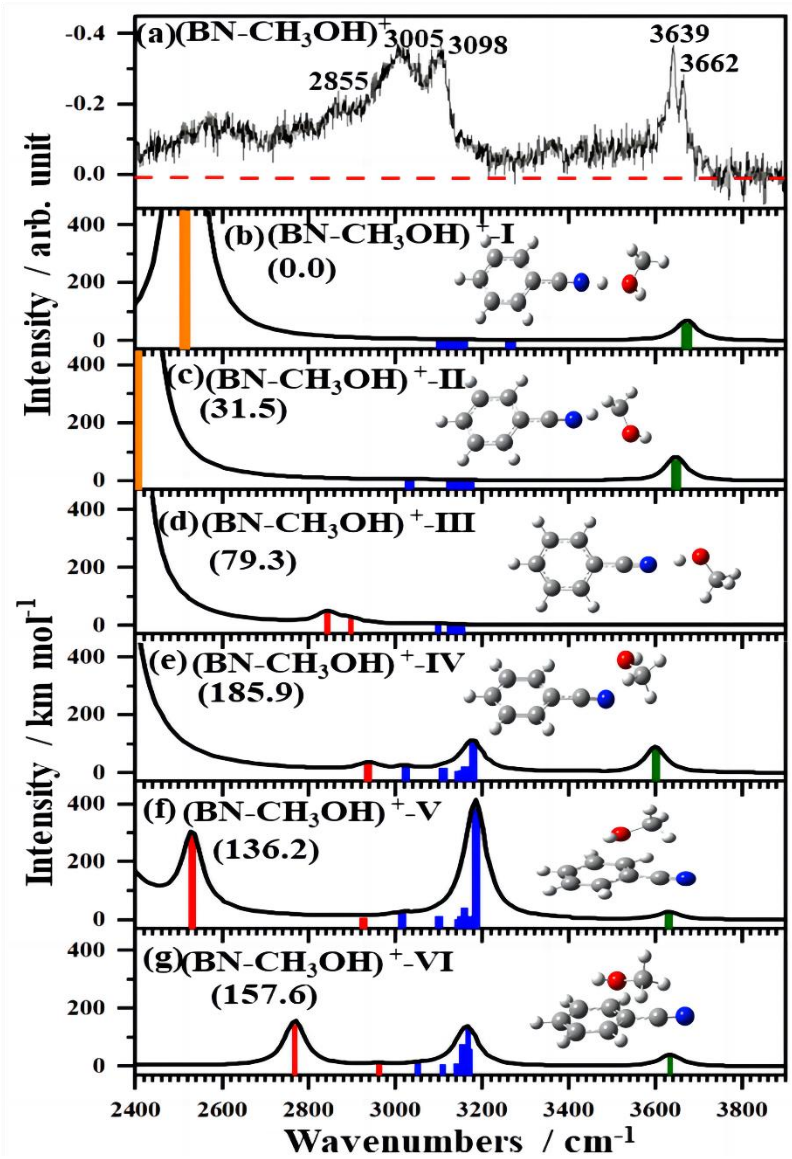

Figure S7. (a) Experimental and (b)-(g) calculated IR spectra of $(BN\text{-}CH_3OH)^+$ in the 2400−3900 cm^{-1} region. Calculated spectra with a scaled factor of 0.96 under MP2/aug-cc-pVDZ method, and the convoluted spectra generated by a Lorentzian line shape function with a width of 30 cm^{-1} (FWHM). The predicted structures with relative energy at the zero point vibrational level (the number in parentheses in kJ mol^{-1}) are also shown. The orange, red, blue and green sticks represent the proton-shared NH stretching vibration, CH stretching vibration of C (sp^3)–H and C (sp^2)–H, and OH stretching vibration, respectively.

Table S1. Comparison of observed and scaled harmonic frequencies of (BN-CH_3OH) and their assignments.

Experiment	Calculation[a]					Assignments
Bands (cm^{-1}) [b]	**(BN-CH_3OH)-I**	**(BN-CH_3OH)-II**	**(BN-CH_3OH)-III**	**(BN-CH_3OH)-IV**	**(BN-CH_3OH)-V**	
2837	2880(40.8)[c]	2880(74.2)	2860(65.5)	2878(32.7)	2888(42.1)	s[d]-CH_3-stretch
2917	2935(51.9)	2934(46.6)	2905(60.4)	2932(32.6)	2946(35.0)	
2949	2992(22.9)	2992(26.1)	2978(43.9)	3000(32.4)	3005(19.1)	a[e]-CH_3-stretch
2982						
3036	3053(2.9)	3053(1.6)	3056(0.0)	3060(0.0)	3053(2.1)	a-CH-stretch
	3063(20.3)	3064(14.9)	3066(3.9)	3068(2.8)	3066(9.0)	
3068	3068(2.0)	3071(8.0)	3073(8.1)	3076(5.3)	3079(4.6)	s-CH-stretch
	3075(17.0)	3078(17.1)	3082(6.0)	3083(4.2)	3082(6.4)	
	3082(9.0)	3081(14.5)	3085(3.4)	3087(2.5)	3093(0.8)	
3630	3621(55.3)	3611(118.9)	3572(210.0)	3665(74.0)	3676(27.1)	H bond-stretch

[a] calculated at B3LYP-D3(BJ)/aug-cc-pVDZ level and scaled by a factor of 0.96. [b]wavenumbers of the bands in the IR spectra of (BN-CH_3OH). [c] the value in the parentheses is IR intensities in km mol^{-1}. [d] symmetric stretching vibration. [e] antisymmetric stretching vibration.

Table S2. Comparison of observed and scaled harmonic frequencies of $(BN\text{-}CH_3OH)^+$ and their assignments.

Experiment	Calculation[a]						Assignments
Bands (cm^{-1}) [b]	**$(BN\text{-}CH_3OH)^+$-I**	**$(BN\text{-}CH_3OH)^+$-II**	**$(BN\text{-}CH_3OH)^+$-III**	**$(BN\text{-}CH_3OH)^+$-IV**	**$(BN\text{-}CH_3OH)^+$-V**	**$(BN\text{-}CH_3OH)^+$-VI**	
broad feature	2474(7711.7)[c]	2334(6139.6)					Photon-shared N···H···O or N···H···C stretch
2855			2816(240.5)<br>2827(24.3)	2897(48.6)	2869(52.7)	2925(10.7)	s[d]-CH_3-stretch
3005		2990(6.1)		2982(16.3)	2962(0.7)		a[e]-CH_3-stretch
3098	3065(2.0)<br>3093(0.1)<br>3100(0.2)<br>3104(0.2)<br>3112(0.6)<br>3116(0.02)<br>3221(0.1)	3093(0.01)<br>3101(0.2)<br>3104(0.5)<br>3113(0.9)<br>3116(0.01)<br>3125(0.2)	3045(75.2)<br>3095(0.1)<br>3103(0.1)<br>3106(0.4)<br>3114(1.6)<br>3118(4.1)	3074(17.9)<br>3095(0.001)<br>3105(0.1)<br>3108(2.0)<br>3116(5.2)<br>3119(0.6)	3061(13.8)<br>3096(0.1)<br>3104(1.2)<br>3108(3.1)<br>3116(7.3)<br>3119(2.6)	3006(16.9)<br>3066(3.3)<br>3097(0.1)<br>3104(1.0)<br>3112(3.1)<br>3118(5.5)<br>3122(2.7)	a-CH-stretch<br><br>s-CH-stretch
3639		3641(76.9)		3602(342.7)	3629(96.3)	3631(93.4)	free OH-stretch
3662	3669(56.3)						free OH-stretch

[a] calculated at B3LYP-D3(BJ)/aug-cc-pVDZ level and scaled by a factor of 0.967. [b]wavenumbers of the bands in the IR spectra of $(BN\text{-}CH_3OH)^+$. [c] the value in the parentheses is IR intensities in km mol^{-1}. [d] symmetric stretching vibration. [e] antisymmetric stretching vibration.

Table S3. Comparison of relative energies of $(BN\text{-}CH_3OH)^+$ clusters in Figure 3 under different three methods.

	Relative energy[a] (kJ mol^{-1})					
**Methods**	**$(BN\text{-}CH_3OH)^+$-I**	**$(BN\text{-}CH_3OH)^+$-II**	**$(BN\text{-}CH_3OH)^+$-III**	**$(BN\text{-}CH_3OH)^+$-IV**	**$(BN\text{-}CH_3OH)^+$-V**	**$(BN\text{-}CH_3OH)^+$-VI**
**B3LYP-D3(BJ)/aug-cc-pVDZ**	**0**	**15.1**	**28.1**	**39.3**	**42.8**	**46.8**
**M06-2x/aug-cc-pVDZ**	**0**	**27.1**	**55.4**	**78.0**	**66.0**	**68.4**
**MP2/aug-cc-pVDZ**	**0**	**31.5**	**79.3**	**185.9**	**136.2**	**157.6**

[a] The relative energy of $(BN\text{-}CH_3OH)^+$-I all set to 0 kJ mol^{-1} under the three methods.
